# Cigarette Smoking as a Predictor of Male DUI Recidivism

**DOI:** 10.3390/ijerph182010761

**Published:** 2021-10-14

**Authors:** Claudio Terranova, Giovanni Forza, Elena Beccegato, Angelo Ruggeri, Guido Viel, Alessia Viero, Massimo Montisci

**Affiliations:** Legal Medicine and Toxicology, Department of Cardiac, Thoracic, Vascular Sciences and Public Health, University of Padova, Via G. Falloppio n.50, 35121 Padova, Italy; giovanni.forza@aopd.veneto.it (G.F.); beccegato.elena@gmail.com (E.B.); dr.angeloruggeri@gmail.com (A.R.); guido.viel@unipd.it (G.V.); alessia.viero@aopd.veneto.it (A.V.); massimo.montisci@unipd.it (M.M.)

**Keywords:** driving under the influence, recidivism, tobacco use, alcohol impairment, medico-legal ascertainment

## Abstract

This study aimed to investigate the predictors of recidivism in first-time driving under the influence (DUI) offenders, analyzing variables derived from medico-legal and toxicological examinations. The research was structured as a comparative study for the period 2012–2019. DUI offenders with a blood alcohol concentration >0.5 were included in the study. The case group consisted of recidivist offenders, while the comparison group consisted of first-time offenders. Personal data, socioeconomics, and parameters linked to the DUI were compared between the two groups. Significance was determined by chi-square and Mann–Whitney tests. To prevent confounding effects, multivariate binary logistic regression analysis was performed. Our sample encompassed 1678 subjects (196 in the case group, 1482 in the comparison group). Gender, driving license category, education, and tobacco use resulted in significant differences between the groups. In a model including age at DUI, education, and smoking habit as independent variables, higher educational levels (high school, bachelor’s) and older age protected against recidivism, whereas smoking >20 cigarettes/day was an independent risk factor for recidivism. Recidivist offenders have specific characteristics indicating different therapeutic programs and carefulness in driving license regranting. A higher tobacco consumption in recidivists suggests that the use of this substance could influence the risk of DUI for reasons that will need to be explored.

## 1. Introduction

Driving under the influence (DUI) of alcohol causes thousands of deaths in traffic accidents each year worldwide [1,2,3,4]. The United States reported 10,511 alcohol-related driving fatalities in 2018, accounting for 29% of all road fatalities that year [5]. In the European Union, alcohol-related road fatalities were estimated to account for 25% of all road deaths (25,150) in 2018 [6]. In Italy, 3692 individuals were injured in alcohol-related crashes in 2019, accounting for 9.2% of road accidents with injuries [7]. DUI subjects are heterogeneous [8] in relation to demographic, socioeconomic, and psychopathological variables. The misuse of psychoactive substances other than alcohol [9] among DUI subjects is another factor that increases the complexity and variability of this population. The simultaneous use of alcohol and drugs increases the risk of being involved in a road accident [10] even if many variables should be considered in the analysis of this relation. A particular subgroup of DUI subjects is represented by recidivist offenders who, following the first DUI episode, relapse to driving while intoxicated or impaired according to established legal limits [8,11].

The early identification of DUI recidivism could be important for preventing recurrent DUI behavior in subjects at higher risk of being involved in crashes than first-time offenders [11] and considered responsible for a significant proportion of all DUI offenses [12].

The understanding of risk factors for drunk driving recurrence has been used to develop preventive strategies through judicial and/or clinical actions. Judicially, approaches based on the theory of deterrence or social control have been developed [11,13]. The first is based on the belief that antisocial behavior can be discouraged through the threat of punishment on a general level (e.g., legal limits for DUI) or on a specific level (e.g., heavy fines, license suspension); the second considers that legal factors (e.g., jail time, license suspension) and extralegal factors (e.g., moral commitment, group support, etc.) can prevent recidivism by inducing the subject to conform [11].

The different psychological, psychiatric, social, and environmental characteristics of recidivist offenders require different therapeutic approaches [11]. Psychologically, rehabilitation or prevention of recidivist offenders can be based, for example, on the cognitive-behavioral model [14]. Environmental factors could be represented by vehicle ignition interlock devices [15].

Many studies have focused on establishing significant predictors of DUI relapse; demographic characteristics, criminal history, alcohol and drug-related factors, personality traits [16,17] (e.g., sensation seeking behaviors or propensity to hostility while driving), and psychiatric features [18] have each been studied, with mixed results for some variables [11].

Repeat DUI offenders are usually males [19]; repeat female DUI offenders are less frequently observed, and these data suggest that risk factors are different for women and men [20]. Results are discordant in studies comparing the age of recidivist offenders [19,21]; recidivist offenders were older than first-time offenders according to some authors [11], while in other studies, recidivists were reported to typically be younger than 30 [22]. Low education, low income or unemployment, and socioeconomic disadvantage have been associated with DUI recidivism [23,24,25]. It has also been observed that DUI recidivism among males is associated with divorce, separation, or widowhood [11]. This has not been confirmed in female DUI recidivists [26].

Recidivist offenders are also more deviant and have higher levels of psychiatric distress and substance abuse [17]. The importance of substance use disorders has been confirmed by the use of assessment instruments containing references to substance use disorder classifications [27]. Moreover, the co-use of alcohol and other psychoactive substances may be a condition related to a higher risk of DUI recidivism [28]. According to Roberts and Fillmore [29], recidivism cannot be entirely attributed to alcohol use disorder [29], and thus, other factors could play a role.

Among substance use disorders, smoking was associated with drinking and driving [30], at-risk alcohol use [31], and greater risk of injury [32]. Cigarette smoking could share with DUI recidivism and sensation seeking a common mechanism represented by dysregulation in the hypothalamic–pituitary–adrenal axis [33].

The relationship between blood alcohol concentration (BAC) at the time of the offense and DUI recidivism has been extensively analyzed, with differing results. Some authors [34,35,36] found a positive correlation, but other researchers found no or only non-significant associations [21,37]. A refusal to submit to blood alcohol analysis was associated in some studies with a risk of recidivism [38]. Finally, involvement in road accidents is a confirmed risk factor for DUI recidivism [24].

Considering the different, sometimes confounding, factors related to DUI recidivism, it could be of interest to contribute to the analysis of the phenomenon by means of collecting objective data. Thus, the aim of the present study was to investigate the predictors of DUI recidivism among first-time DUI offenders among an Italian population, analyzing variables derived from medico-legal and toxicological examinations.

## 2. Materials and Methods

The research was structured as a comparative study for the period 2012–2019. Drivers with an alcohol-related DUI according to Italian legislation (the BAC limit in Italy is 0.5 g/dL) were included in the study. According to Italian legislation, these drivers must be examined before their driver’s licenses are reinstated. The population study was thus represented by subjects examined at the Unit of Legal Medicine and Toxicology, Hospital University of Padova for driving license regranting. Examination was carried out by means of an integrated methodological approach that included the following phases: (1) demographic data collection (age at DUI, gender, driving license category); (2) analysis of documentation regarding the DUI episode (BAC, road accident involvement, presence of psychoactive substances other than alcohol) and the alteration of specific blood markers of chronic alcohol intake; (3) direct examination, including anamnestic data and objective examination; and (4) toxicological analysis, including urine and hair samples. After the examination during which hair samples (with a length of 3–6 cm) were taken, the subjects were monitored for two months through urine samples. The examined subjects were considered either fit or unfit to drive based on an integrated evaluation of the results of the methodological approach according to a protocol adopted in our unit. In particular, excessive alcohol intake was considered a cause for an unfitness to drive classification; the finding of illicit psychoactive substances on hair or urine samples was another cause for the determination of unfitness to drive.

Inclusion criteria were subjects older than 17 years who had at least one episode of DUI with BAC over 0.5 g/L during the study period and who were declared fit to drive after the first medico-legal and toxicological examinations. Exclusion criteria were one or more episodes of DUI related to psychoactive substances other than alcohol and/or meeting the criteria of the Diagnostic and Statistical Manual of Mental Disorders [39] for psychiatric disorders, including substance-related and addictive disorders, with the exception of tobacco use disorder. Neurological and medical disorders and age younger than 18 were also exclusion criteria. Also excluded from the study were subjects declared unfit to drive during the first medico-legal and toxicological examination. Finally, subjects declaring tobacco use other than cigarettes were excluded from the study.

Participants were then subdivided into two groups according to the presence of DUI recidivism. Cases were subjects who, after being considered fit to drive, were involved in at least a second episode of DUI. The comparison group included subjects considered fit to drive with no further DUI episodes during the study period.

The two groups were analyzed in relation to the parameters collected during the examination and listed in Table 1.

The parameters were subdivided in classifications of personal data, including age at DUI, gender, driving license category, and tobacco use; socioeconomic conditions, encompassing education, marital status, employment situation, and tobacco use; and parameters linked to DUI, including BAC, road accident involvement, and concurrent substance use other than alcohol at the time of the DUI (see Table 1). For the recidivist group, the lag time between the first and the second DUI episode was collected.

The anonymized data were entered into an Excel spreadsheet, and descriptive analyses were performed for the two groups. The case group was compared to the comparison group according to the variables listed above using a chi-square test. The same analysis was performed to compare males and females. The Mann–Whitney U test was used for continuous data with a nonparametric distribution. The variables that differed significantly between the two groups (*p* < 0.05) in the preliminary analysis were then included in a multivariate binary logistic regression model to prevent spurious effects [40], with significance set at 0.05. Moreover, we analyzed possible predictors of the lag time between the first and the second DUI episode using Cox-regression analysis. Procedures of the study were in accordance with the Declaration of Helsinki of 1975, as revised in 1983.

## 3. Results

The study included 1678 subjects (1485 males and 193 females). The cases numbered 196 (11.7% of the total), with 186 males (94.9%) and 10 females (5.1%). Subjects in the comparison group were 1482 (88.3% of the total), with 1299 males (87.7%) and 183 females (12.3%). The parameters of gender, driving license category, education, employment situation, marital status, tobacco use, age at DUI, current substance use other than alcohol at the time of DUI, BAC at DUI, and road accident involvement, both overall and according to cases versus the comparison group, are provided in Table 2.

The proportions of male subjects and of driving license category type 2 were higher in cases than in the comparison group. Age at DUI resulted in a significant difference between cases and the comparison group, with recidivist offenders younger than one-time DUI offenders (Table 2). Interestingly, tobacco use was more frequent in cases than in the comparison group. In particular, heavy smokers (>20 cigarettes/day) were more prevalent among cases than the comparison group.

Among socioeconomic factors, more subjects with a low educational level were observed in cases than in the comparison group. Occupation, marital status, presence of psychoactive substances other than alcohol at DUI, BAC at DUI, and road accident involvement at DUI were not different between the two groups. Due to the low number of female subjects among cases, further analyses were conducted on male subjects only. As shown in Table 3, significant differences between cases and the comparison groups were confirmed for age at DUI, education, and tobacco use among males. Any difference in driving license category was not confirmed among males. An inverse correlation was found between the probability of recidivism and educational level (Spearman’s rho coefficient −0.08, *p* = 0.002).

Age at DUI, education (8 and 13 years of education and degree), and smoking habits (more or fewer than 20 cigarettes per day) were included in the logistic regression model as independent variables. Higher educational levels (high school degree, *p* = 0.002; bachelor’s degree, *p* = 0.006), and older age (*p* < 0.001)) were protective against recidivism, whereas smoking more than 20 cigarettes/day resulted in an independent risk factor for recidivism (*p* = 0.025) (Table 4).

The role of cigarette smoking in recidivist was further explored based on the results of Table 4, drawing attention to tobacco use. We combined the risk factors “heavy smoker”—“low education” and the risk of recidivism increased by 1.9 times (OR 1.92, 95% CI 1.32–2.81). We further combined heavy smoking, education, and age. Being heavy smokers under the age of 30 with low education increased the risk by 2.7 times (OR 2.70, 95% CI 1.61–4.54, *p* > 0.001).

Smoking more than 20 cigarettes was evaluated in relation to BAC. Subjects who were heavy smokers with BAC > 1.5 g/L were not at higher risk of recidivism compared with subjects who were only heavy smokers (OR 1.543, 95% CI 0.79–3.00, *p* = 0.19).

Finally, by a Cox-regression model including factors associated with recidivism, we did not identify any predictor of a shorter lag time between the first and the second DUI episode (*p* = 0.54).

## 4. Discussion

Investigating the predictors of DUI recidivism contributes significantly to traffic injury prevention because recidivist offenders exhibit risky driving behavior more frequently than one-time DUI offenders [11]. The importance of this study lies in the methodological approach. The availability of medico-legal and toxicological data allowed us to reduce the bias related to an examination based solely on reported data; in particular, data on DUI episodes and the use of psychoactive substances other than alcohol during the medico-legal evaluation were available. Personal data, socioeconomic factors, and elements related to DUI were investigated with the aim of finding distinguishing features in recidivist offenders.

As the number of female recidivist offenders in our sample was too low, we focused our attention primarily on males. Most of our results deriving from comparisons between the two groups were confirmed in males (with the exception of driving license) but it was not possible to repeat the result for females due to the distribution of the considered variables or the low number of female subjects.

Recidivist offenders were younger than first-time offenders, which is partly consistent with previous research [22]. Our results suggest that an older age is associated with lower odds of being a recidivist offender and that an older age decreases the odds of being a recidivist offender by 5% compared to a younger age. The mean age in both cases and comparison subjects was low in our study, thus the majority of our subjects could be considered young at high risk in other studies [22]. 

Driving license category distribution in males was not different between the two groups. No female subjects possessed a driving category to drive heavy vehicles, and this fact is responsible for the apparent significant difference between the two groups when analyzing the overall sample.

The most important result of our study was the significant difference between recidivist and first-time DUI offenders in terms of tobacco use. Recidivists reported consuming more than 20 cigarettes per day significantly more frequently than first-time offenders. The threshold of 20 cigarettes per day could be considered an indirect sign of heavy smoking, as such consumption over a period of 10–20 years is associated with a clinically relevant increase in morbidity [41]. Heavy smoking could indicate a tobacco use disorder and a possible association with other substance use disorders. Our results suggest that heavy smoking may be a predictor of risky alcohol intake leading to DUI. The consumption of more than 20 cigarettes per day was associated with higher odds of being a recidivist. Some factors may explain this finding. A more severe form of tobacco use disorder could be related to a diagnosis of alcohol use disorder [42]. Tobacco use disorder may also be associated with impaired and risky decision-making [43]. Thus, DUI recidivism could be considered a consequence of impaired and risky decision-making rather than an alcohol use disorder. This interpretation is consistent with empirical observations [44,45] that smokers take greater job risks than nonsmokers but receive less hazard pay. This choice, which appears irrational and in contrast with the model of compensating differentials, may be motivated by the fact that smokers exhibit risk-taking behavior patterns in various aspects of their lives, including driving under the influence of substances or taking greater job risks. Tobacco use might decrease the subjective intoxicating and sedating effects of alcohol, leading to heavier drinking episodes [42]. Finally, tobacco use while driving has been associated with road accidents [46,47]. Independent of the aim of the study, it is interesting to highlight that the prevalence of tobacco use disorder in our samples (cases and the comparison group) was almost three times that observed in the overall Italian population [48]. This is consistent with the fact that alcohol and tobacco use are highly concurrent [42,49]. 

The education level data showed a prevalence of basic education levels in recidivist offenders (45.9%, compared to 34% of first-time offenders). Our results suggest that a high school diploma and a degree reduce the odds of being a recidivist by 55% and 65%, respectively, compared to basic education levels. This is consistent with the literature [50], although an epidemiological evaluation cannot establish a causal relationship between education level and drunk driving. However, as previously suggested [50], the relationship between educational underachievement and drunk driving is similar to that between low educational levels and deviance [50,51]. No differences were observed for employment. Other studies showed a correlation between unemployment, lower income, and recidivist offenses [11]. Being unmarried, divorced, separated, or widowed have also been related to recidivism [11]. However, such relationships were not found in our sample, perhaps due to social changes in Western countries over the last 30 years, with a general reduction in marriage rates. 

Unexpectedly, we found that the concurrent use of psychoactive substances was not associated with recidivism in our sample. This can be due to the low prevalence of psychoactive substance use in our cohort, which also did not allow us to evaluate the effect of the different single types of substances. No differences were observed in BAC levels and road accident involvement. Therefore, these parameters did not predict a risk of recidivism in our population. The lack of an association between BAC and recidivism is consistent with previous studies [20,36] and is probably due to the characteristics of the studied population, which included few subjects with an alcohol use disorder.

The lack of a significant difference between the two groups in terms of road accident involvement, which is an element linked to recidivist offenders [24], was probably due to the low prevalence of individuals with concurrent drug use at DUI. While some psychoactive substances have been associated with a higher risk of being involved in a road accident [10], for other substances, the relationship is exceedingly complex, and no conclusions can be drawn [10].

An analysis of the presence of people injured during accidents and the time of road accidents could provide useful data for differentiating the two groups: it is possible that the presence of people injured during a road accident could act as a deterrent against recidivism.

The integrated analysis of possible risk factors showed an interaction between smoking, age, and education, which increased the risk of belonging to the group of recidivists. Surprisingly, subjects who were heavy smokers plus BAC > 1.5 were not at higher risk of recidivism compared with subjects who were only heavy smokers, reinforcing our result that smoke is an important predictor of recidivism.

### 4.1. Medico-Legal Repercussions and Preventive Measures

The results of our study, specifically concerning nicotine use, should be taken into account in the medicolegal and clinical fields. The medicolegal approach to assessing DUI offenders’ fitness to drive should consider many aspects related to smoking habits. Tobacco use should be investigated in terms of quantity, type, and frequency. A temporal relationship between smoking and being involved in a road accident should also be investigated. The results of these assessments could determine the duration of the period of fitness to drive acknowledged by the evaluating units or indicate an opportunity for monitoring programs.

Preventive measures for individuals considered at higher risk could include specific awareness campaigns. Preventive education programs for DUI offenders should focus not only on the effects of alcohol but also on those of nicotine use on driving ability [12]. Such programs could also address potential substance use disorders, including tobacco use disorder, therapeutically.

### 4.2. Limits of the Study and Future Directions

The present study has some limitations. The first limitation is the potential erroneous inclusion of a subject in the control group. Although the period analyzed was broad, it cannot be excluded that a subject in the control group could have become a recidivist offender at a later date, after the conclusion of the study. The second limitation of the study is the low number of female recidivist offenders. This element prevented further gender-related analysis.

Possible future developments of the study include the collection of additional data relating to road accidents. The injury or death of people in road accidents could influence the risk of DUI recurrence. The time of the felony and/or road accident could be another important element in the evaluation of DUI offenders. Finally, the association of the consumption of different specific psychoactive substances at the time of DUI could provide further important elements regarding the risk of recurrence of the crime.

## 5. Conclusions

The present study confirms that recidivist offenders have specific characteristics that indicate the use of different therapeutic programs and carefulness in driving license regranting. A methodological approach should encompass not only medico-legal and toxicological data but also an evaluation of subjects’ behavioral features. The increased tobacco consumption among repeat offenders could have behavioral consequences or indicate the risk of another substance use disorder. The causes of these differences need to be clarified. This data is very important for either the evaluation of a subject with a previous DUI or in evaluating subjects for a tobacco problem.

Gender differences need to be investigated in a larger sample, but the data suggest different risk factors in male and female recidivists, and furthermore, these data imply different therapeutic programs should be used. Other data, such as education, age at DUI, and type of license, require specific and in-depth studies aimed at clarifying their meaning.

## Figures and Tables

**Table 1:** Data collected via the study form.

Variable	
Personal Data	
**Age at DUI**	
**Gender**	Female
	Male
**Driving license category**	Type 1 *
	Type 2 *
**Tobacco use**	No use
	Less than 20 cigarettes per day
	More than 20 cigarettes per day
**Socioeconomic Factors**	
**Education**	5 years
	8 years
	13 years–high school degree
	Bachelor’s degree
**Employment situation**	Employed
	Freelance
	Insecure employment
	Unemployed
	Student
**Marital status**	Single
	Married
	Divorced
	Widower/widow
**Driving under the Influence Variables**	
**DUI**	Alcohol only
	Alcohol plus psychoactive substances
**BAC at DUI**	0.5–0.8 g/L
	0.8–1.5 g/L
	1.5–2.5 g/L
	>2.5 g/L
	Refusal of alcohol determination
**Road accident at DUI?**	Yes
	No

Notes: DUI = Driving under the influence. * Type 1 category permits driving cars or motorcycles. Type 2 category permits driving a 3.5 vehicle or more.

**Table 2:** Personal data, socio-economic factors, and conditions at DUI in cases and the comparison group.

Variable		Total N = 1678 (100%)	Cases N = 196 (100%)	Comparison Subjects N = 1482 (100%)	*p*-Value *
Personal Data					
**Age at DUI**, years, mean (Standard deviation)		32.58 (9.667)	30.56 (8.96)	32.85 (9.72)	**0.001**
**Gender**	Female, n (%)	193 (11.5)	10 (5.10)	183 (12.34)	**0.003**
	Male, n (%)	1485 (88.5)	186 (94.89)	1299 (87.65)	
**Driving licence category** type 1 **		1519 (90.52)	168 (85.71)	1351 (91.16)	**0.034**
Driving licence category type 2		155 (9.23)	26 (13.26)	129 (8.70)	
**Tobacco use** **					
No use		527 (31.40)	58 (29.59)	469 (31.64)	**0.002**
Less than 20 cigarettes		922 (54.94)	96 (49.97)	826 (55.73)	
More than 20 cigarettes		226 (13.46)	42 (21.42)	184 (12.41)	
**Socio-Economic Factors**					
**Education** **					
5 years		35 (2.08)	3 (1.53)	32 (2.15)	**0.003**
8 years		558 (33.25)	87 (44.38)	471 (31.78)	
13 years		879 (52.38)	91 (46.42)	788 (53.17)	
Degree		202 (12.03)	15 (7.65)	187 (12.61)	
**Employment Situation** **					
Employed		1095 (65.25)	127 (64.79)	968 (65.31)	0.106
Freelance		337 (20.08)	47 (24.97)	290 (19.56)	
Insecure employment		14 (0.83)	0 (0)	14 (0.94)	
Unemployed		144 (8.58)	18 (9.18)	126 (8.50)	
Student		85 (5.06)	4 (2.04)	81 (5.46)	
**Marital status** **					
Single		1027 (61.20)	127 (64.79)	900 (60.72)	0.667
Married		505 (30.09)	55 (28.06)	450 (30.36)	
Divorced		133 (7.92)	13 (6.63)	120 (8.09)	
Widower/widow		5 (0.29)	1 (0.51)	4 (0.26)	
**Driving under the Influence Variables**					
**DUI–Alcohol**		1585 (94.45)	189 (96.42)	1396 (94.19)	0.199
DUI Alcol plus psychoactive substances		93 (5.54)	7 (3.57)	86 (5.80)	
**BAC at DUI**					
0.5–0.8 g/L		387 (23.06)	40 (20.40)	347 (23.41)	0.475
0.8–1.5 g/L		650 (38.73)	83 (42.34)	567 (38.25)	
1.5–2.5 g/L		356 (21.21)	46 (23.46)	310 (20.91)	
>2.5 g/L		53 (3.15)	6 (3.06)	47 (3.17)	
Refusal of alcohol determination		232 (13.82)	21 (10.71)	211 (14.23)	
**Road accident at DUI** **		371 (22.10)	45 (22.95)	326 (21.99)	0.705
No road accident		1305 (77.77)	149 (76.02)	1156 (78.0)	

Abbreviations: Driving under the influence (DUI). * *p*-value refer to chi-square test for dichotomous variables and to Mann-Whitney test for continuous data with non-parametric distribution. *p-*values < 0.05 were highlighted in bold. ** Data may be incomplete for some subjects. The sum of the numbers and of the percentages with reference to the variable considered may not correspond to the total or to 100%.

**Table 3:** Personal data, socio-economic factors, and conditions at DUI in male recidivists and comparison subjects.

Variable	Total N = 1485 (100%)	Cases N = 186 (100%)	Comparison Subjects N = 1299 (100%)	*p*-Value *
Personal Data				
**Age at DUI**, years, mean (Standard deviation)	32.90 (9.75)	30.81 (8.98)	33.20 (9.82)	**0.02**
**Driving licence category** type 1 **	1328 (89.42)	160 (86.02)	1168 (89.91)	0.093
Driving licence category type 2	155 (10.43)	26 (13.97)	129 (9.93)	
**Tobacco use** **				
No use	472 (31.78)	54 (29.03)	418 (32.17)	**0.005**
Less than 20 cigarettes	801 (53.93)	91 (48.92)	710 (54.65)	
More than 20 cigarettes	211 (14.20)	41 (22.04)	170 (13.08)	
**Socio-Economic Factors**				
**Education** (5 years) **	34 (2.28)	3 (1.61)	31 (2.38)	**0.007**
Education—(8 years)	524 (35.28)	86 (46.23)	438 (33.71)	
Education—(13 years)	765 (51.51)	84 (45.16)	681 (52.42)	
Education—(degree)	160 (10.77)	13 (6.98)	147 (11.31)	
**Employment Situation**—Employed **	977 (65.79)	122 (65.59)	855 (65.81)	0.129
Freelance	315 (21.21	47 (25.26)	268 (20.63)	
Insecure employment	14 (0.94)	0 (0)	14 (1.07)	
Unemployed	115 (7.74)	14 (7.52)	101 (7.77)	
Student	63 (4.24)	3 (1.61)	60 (4.61)	
**Marital status** Single **	895 (60.26)	119 (63.97)	776 (59.73)	0.617
Married	457 (30.77)	53 (28.49)	404 (31.10)	
Divorced	122 (8.21)	13 (6.98)	109 (8.39)	
Widower/widow	4 (0.26)	1 (0.53)	3 (0.23)	
**Driving under the Influence Variables**				
**DUI–Alcohol**	1397 (94.07)	180 (96.77)	1217 (93.68)	0.095
DUI Alcol plus psychoactive substances	88 (5.92)	6 (3.22)	82 (6.32)	
**BAC at DUI**				0.406
0.5–0.8 g/L	326 (21.95)	38 (20.43)	288 (21.17)	
0.8–1.5 g/L	568 (38.24)	80 (43.01)	488 (37.56)	
1.5–2.5 g/L	326 (21.95)	43 (23.11)	283 (21.78)	
>2.5 g/L	47 (3.16)	5 (2.68)	42 (3.23)	
Refusal of alcohol determination	218 (14.68)	20 (10.75)	198 (15.24)	
**Road accident at DUI** **	322 (21.68)	39 (20.96)	283 (21.78)	0.856
No accident	1161 (78.18)	145 (77.95)	1016 (78.21)	

Abbreviations: Driving under the influence (DUI). * *p*-value refer to chi-square test for dichotomous variables and to Mann-Whitney test for continuous data with non-parametric distribution. *p*-values < 0.05 were highlighted in bold. ** Data may be incomplete for some subjects. The sum of the numbers and of the percentages with reference to the variable considered may not correspond to the total or to 100%.

**Table 4:** *p*-Value, Odds ratio, and Confidence interval of the variables associated to recidivism using the multiple logistic regression model.

Variable	*p*-Value	OR *	95% CI **
**Education** ***	<0.001		
Education—(8 years)	0.421	0.794	0.452–1.393
Education—(13 years)	0.002	0.451	0.271–0.753
Education—(degree)	0.006	0.355	0.169–0.744
**Tobacco** ****	0.007		
Less than 20 cigarettes	0.388	0.857	0.603–1.218
More than 20 cigarettes	0.025	1.676	1.066–2.635
**Age at DUI**	<0.001	0.958	0.944–0.972

* OR = Odds ratio. ** CI = Confidence interval. *** Reference category: 5 years. **** Reference category: no tobacco use.

## Data Availability

The datasets generated and analyzed during the current study will not be publicly available due to privacy and confidentiality reasons.
